# Dual-Acting Nitric Oxide Donor and Phosphodiesterase Inhibitor TOP-N53 Increases Lifespan and Health Span of *Caenorhabditis elegans*

**DOI:** 10.17912/micropub.biology.001090

**Published:** 2024-04-10

**Authors:** Martina Rudgalvyte, Paola Atzei, Rita de Brito Francisco, Reto Naef, Dominique A. Glauser

**Affiliations:** 1 Dept. Biology, University of Fribourg, Chemin du Musée 10, 1700 Fribourg, Switzerland; 2 TOPADUR Pharma AG, Grabenstrasse 11A, 8952 Schlieren, Switzerland

## Abstract

The quest for extending lifespan and promoting a healthy aging has been a longstanding pursuit in the field of aging research. The control of aging and age-related diseases by nitric oxide (NO) and cGMP signaling is a broadly conserved process from worms to human. Here we show that TOP-N53, a dual-acting NO donor and PDE5 inhibitor, can increase both lifespan and health span in *C. elegans* .

**Figure 1. TOP-N53 extends C. elegans lifespan and health span:**
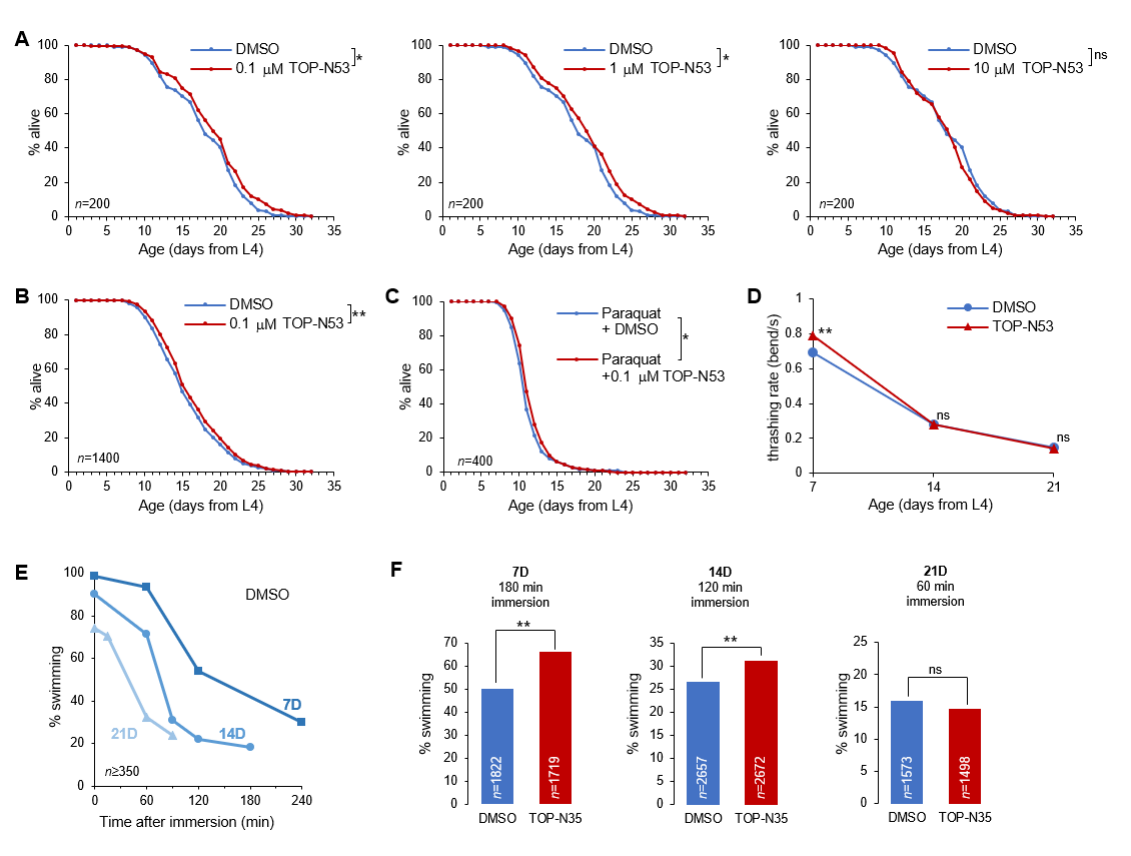
Lifespan curves for worms with the indicated treatments ( **A-C** ). TOP-N53 dose response ( **A** ), confirmation of the beneficial effect of 0.1 μM TOP-N53 ( **B** ) and impact of 0.1 μM TOP-N53 in condition of oxidative stress with 6 mM paraquat ( **C** ). Analysis of age-dependent decline of swimming speed ( **D** , *n* ≥ 1498) and endurance ( **E-F** ). Progressive decline in endurance during aging in DMSO control worms, as a measure of healthiness decline ( **E** ) and beneficial effect of 0.1 μM TOP-N53 to delay the aging-evoked swimming endurance decline ( **F** ). *, *p* <.05; **, *p* <.001; ns, not significant; *n* , number of scored individuals per condition.

## Description

Nitric oxide (NO) is a conserved signaling molecule, controlling diverse cellular functions and physiological processes, including vascular function, immune response, neuronal signaling and aging (Lundberg and Weitzberg 2022) . NO activates soluble guanylyl cyclases (sGC), which in turn produce the cyclic guanosine-monophosphate (cGMP) second messenger. cGMP signaling is terminated by its degradation by phosphodiesterases (PDEs). Whereas high levels of NO may display toxic effects (McCann, Mastronardi et al. 2005) , age-related conditions, such as increased cardiovascular risk, are associated with decreased expression of essential NO signaling components and long-term depletion in cGMP (Sverdlov, Ngo et al. 2014, Cai, Wu et al. 2023) .

TOP-N53 is a first-in class drug, acting as a NO donor and phosphodiesterase 5 (PDE5) inhibitor developed by TOPADUR Pharma AG to treat chronic wounds and presently advancing to clinical phase II (Naef, Tenor et al. 2020) . The TOP-N53 dual mode of action results from its intracellular bio-activation, which produces NO and TOP-52 metabolites. NO promotes cGMP synthesis via sGC, whereas TOP-52 inhibits PDE5 to prevent cGMP degradation. TOP-52 is a very potent PDE5 inhibitor with an IC50 value (0.1 nM) markedly lower than that of its precursor TOP-N53 (1.18 nM) or other marketed PDE5 inhibitors, such as Sildenafil (IC50: 7.68 nM) (Naef, Tenor et al. 2020) . TOP-N53 promotes wound healing in mice (Ben-Yehuda Greenwald, Tacconi et al. 2021) and activates a cytoprotective autophagy pathway in fibroblasts (Martínez-Martínez, Atzei et al. 2022). Its potential to favor a healthy aging is unknown.

Due to its short lifespan, genetic tractability, and the evolutionary conservation of aging-related pathways, *Caenorhabditis elegans* is an attractive model to investigate aging and explore new pharmacological interventions (Kenyon 2005, Ye, Linton et al. 2014, Tissenbaum 2015, Weinkove and Zavagno 2021) . The NO-cGMP signaling pathway controls *C. elegans* longevity. Exogenous supply of NO via NO-producing bacteria, chemical agents or controlled NO-release from nanoparticles modulates worm lifespan (Gusarov, Gautier et al. 2013, Jiang, Cheng et al. 2020) . High NO doses shorten, whereas lower doses extend lifespan. In different contexts, approaches either up-regulating or down-regulating cGMP signaling have been shown to increase lifespan (Hirose, Nakano et al. 2003, Hahm, Kim et al. 2009) . Given the ambivalent effects that either NO or cGMP signaling manipulation can produce on lifespan, we anticipate that dose adjustments will be crucial in future pharmacological interventions. Furthermore, the impact of concomitant modulation on both NO and cGMP is not known.

The objective of this study was to assess the potential of TOP-N53 in promoting a healthy aging in *C. elegans* . We started our investigations by comparing the lifespan of animals treated with different TOP-N53 concentrations (0.1, 1 and 10 μM) or with vehicle (DMSO). Whereas the highest dose (10 μM) had no effect, we observed a ~5% lifespan extension with lower doses (0.1 and 1 μM) ( Fig. 1 A ). It is possible that the highest dose causes some side effects due to high levels of NO and to an excessive inhibition of PDEs.

We pursued our investigations with the lowest TOP-N53 concentration (0.1 μM), as it seemed effective and closer to the concentration expected to be used in human. Because the drug effect was of moderate magnitude, we next ascertained its robustness by conducting six additional independent lifespan experiment replicates, each comparing DMSO with 0.1 μM TOP-N53 (each scoring 200 animals per condition). In 6 out of 7 assays, TOP-N53 extended mean lifespan and when aggregating the data, we could confirm a significant overall increase in mean lifespan from 15.77 to 16.42 days ( *p* =.0007, total number of animals per condition *n* =1400), representing a lifespan extension of ~4% (Fig 1B).

Next, we tested if the TOP-N53 effect could be enhanced when worms are treated with 6 mM paraquat, causing oxidative stress (Bridi, Barros et al. 2015) and slightly shortening animal lifespan ( Fig. 1C ). In the presence of paraquat, TOP-N53 was still effective, but the effect (~ 5% extension of mean lifespan) was similar in magnitude to the situation in the absence of paraquat (Fig 1B, C). We conclude that the drug benefits are still present, but not enhanced under a situation of oxidative stress.

Finally, we wondered whether the TOP-N53-induced lifespan extension in *C. elegans* was linked to a better health span. Like in human, defining good health in worms is challenging and arbitrary (Jushaj, Churgin et al. 2020) . Caffeine-induced lifespan extension was previously shown to be accompanied by improved swimming capabilities during aging, which was considered as an indicator of a prolonged health span (Sutphin, Bishop et al. 2012) . We analyzed swimming in 7-, 14- or 21-day old adult animals (7D, 14D, 21D) measuring their swimming speed ( Fig. 1D ) and their endurance by scoring the fraction of animals having stopped swimming after exhaustion (fraction paralyzed, Fig. 1E and F ). TOP-N53 treatement delayed the age-dependent decline in swimming speed, with higher speed at 7D and similar speed at 14D and 21D as compared to DMSO controls ( Fig. 1D ). In DMSO control, a 50% paralysis was observed after about 120 min for 7D control animals, whereas older worms paralyzed earlier (<90 min and <60 min to reach 50% paralysis in D14 and D21 worms, respectively), which we interpret as another indicator of age-dependent fitness reduction. TOP-N53-treated worms had a better endurance at 7D and 14D, but not at 21D ( Fig. 1E ). Therefore, we conclude that TOP-N53 treatement delays the age-dependent decline in swimming speed and endurance.

Overall, our lifespan assays show that TOP-N53 can extend *C. elegans* lifespan and the maintenance of swimming capabilities suggests a healthy aging. This contrasts with reduced swimming capabilities seen in some long-lived mutants (Bansal, Zhu et al. 2015) . Our findings support the notion that pharmacological interventions targeting the NO-cGMP pathway can produce anti-aging effects *in vivo* , highlight the potential of TOP-N53 toward this goal, and set a useful experimental model to conduct further research to confirm the drug mode-of-action and to further define downstream cellular mechanisms.

## Methods

***Lifespan assays*** *-* All worms were grown at 20°C. N2 worms were synchronized by standard hypochlorite treatment, grown on NGM plates and, at the young adult stage, placed onto NGM plates supplemented with 100 mg/l ampicillin and 40 μM FUDR, and seeded with HT115 *E. coli* (Sutphin and Kaeberlein 2009) . DMSO or TOP-N53 were added to the bacteria suspension prior to seeding the plates, which were left to dry in the dark 24 h prior to experiments. Worms were scored daily for survival and transferred on fresh plates every 5 days or more often if a contamination or a larva was detected. Plates without FUDR were used after the fifth day of adulthood. For oxidative stress experiments, paraquat was added to the growth plates from the young adult stage on and the treatment was maintained until the end of the experiment. Lifespan statistical analyses were performed with the OASIS2 software, using log rank tests to assess differences (Han, Lee et al. 2016) .

***Swimming assays-*** Swimming endurance assays were endpoint experiments using worms treated with DMSO or TOP-N53 exactly as for lifespan assays. Worm swimming was recorded at 25°C. Seven-, 14- or 21-day old adult worms were transferred to 24-well plates, left to recover 1h and then flooded with M9 buffer. Thirty-second videos were recorded, swimming speed was quantified as previously described (Nussbaum-Krammer, Neto et al. 2015) , and, for endurance tests, movies were analyzed manually to score the fraction of paralyzed worms. We performed three independent swimming experiments, each scoring 12 separate populations of at least 15 worms. Two-tailed Student's *t* tests were used to assess speed differences. Fisher's exact tests on the number of swimming/paralyzed worms were used to compare DMSO and TOP-N53 treated worms. Bonferroni corrections were applied to *p* values to compensate for multiple testing.
